# Calibration and Validation of the Dutch-Flemish PROMIS Pain Interference Item Bank in Patients with Chronic Pain

**DOI:** 10.1371/journal.pone.0134094

**Published:** 2015-07-27

**Authors:** Martine H. P. Crins, Leo D. Roorda, Niels Smits, Henrica C. W. de Vet, Rene Westhovens, David Cella, Karon F. Cook, Dennis Revicki, Jaap van Leeuwen, Maarten Boers, Joost Dekker, Caroline B. Terwee

**Affiliations:** 1 Amsterdam Rehabilitation Research Center | Reade, Amsterdam, The Netherlands; 2 Department of Clinical Psychology, The EMGO Institute for Health and Care Research, VU University Medical Center, Amsterdam, The Netherlands; 3 Department of Methodology, The EMGO Institute for Health and Care Research, VU University Medical Center, Amsterdam, The Netherlands; 4 Department of Epidemiology and Biostatistics, The EMGO Institute for Health and Care Research, VU University Medical Center, Amsterdam, The Netherlands; 5 Department of Development and Regeneration, Skeletal Biology and Engineering Research Center, KU Leuven, Louvain, Belgium; 6 Rheumatology, University Hospitals, KU Leuven, Louvain, Belgium; 7 Department of Medical Social Sciences, Northwestern University Feinberg School of Medicine, Chicago, Illinois, United States of America; 8 Outcomes Research, Evidera, Bethesda, Maryland, United States of America; 9 Leones Group BV, Amsterdam, The Netherlands; 10 Department of Rheumatology, VU University Medical Center, Amsterdam, The Netherlands; 11 Department of Rehabilitation Medicine, VU University Medical Center, Amsterdam, The Netherlands; 12 Department of Psychiatry, VU University Medical Center, Amsterdam, The Netherlands; School of Ophthalmology and Optometry and Eye Hospital, Wenzhou Medical University, Wenzhou, Zhejiang, China., CHINA

## Abstract

The Dutch-Flemish PROMIS Group translated the adult PROMIS Pain Interference item bank into Dutch-Flemish. The aims of the current study were to calibrate the parameters of these items using an item response theory (IRT) model, to evaluate the cross-cultural validity of the Dutch-Flemish translations compared to the original English items, and to evaluate their reliability and construct validity. The 40 items in the bank were completed by 1085 Dutch chronic pain patients. Before calibrating the items, IRT model assumptions were evaluated using confirmatory factor analysis (CFA). Items were calibrated using the graded response model (GRM), an IRT model appropriate for items with more than two response options. To evaluate cross-cultural validity, differential item functioning (DIF) for language (Dutch vs. English) was examined. Reliability was evaluated based on standard errors and Cronbach’s alpha. To evaluate construct validity correlations with scores on legacy instruments (e.g., the Disabilities of the Arm, Shoulder and Hand Questionnaire) were calculated. Unidimensionality of the Dutch-Flemish PROMIS Pain Interference item bank was supported by CFA tests of model fit (CFI = 0.986, TLI = 0.986). Furthermore, the data fit the GRM and showed good coverage across the pain interference continuum (threshold-parameters range: -3.04 to 3.44). The Dutch-Flemish PROMIS Pain Interference item bank has good cross-cultural validity (only two out of 40 items showing DIF), good reliability (Cronbach’s alpha = 0.98), and good construct validity (Pearson correlations between 0.62 and 0.75). A computer adaptive test (CAT) and Dutch-Flemish PROMIS short forms of the Dutch-Flemish PROMIS Pain Interference item bank can now be developed.

## Introduction

The prevalence of chronic pain is high in western populations, ranging from 10.1 to 55.2% [1–3]. Chronic pain is defined as pain that persists beyond the normal tissue healing time, in which the most prevalent pain is musculoskeletal pain, with prevalence varying from 30–40% for low back pain, 15–20% for shoulder- and neck pain, 10–15% for chronic widespread pain and 2% for fibromyalgia [3,4]. Chronic pain often leads to substantial limitations in daily activities [4]. Pain interference refers to the degree to which pain interferes with or limits person’s social, mental and physical activities [5]. Self-reported pain interference has increasingly become an important indicator of the experiences of patients with pain and has recently been recommended as a core outcome in international core sets [6,7]. Consequently, pain interference is an important construct to measure in patients with chronic pain.

The National Institutes of Health (NIH) Patient-Reported Outcomes Measurement Information System (PROMIS®) initiative has developed a dynamic assessment system for measuring patient-reported health [8–10]. Included in this system is an item bank that targets pain interference. An item bank is a set of items that measure the same construct and whose parameters have been estimated using an IRT model. Both the item parameters and the person’s parameters are placed on the same underlying metric. Item banks can be used to tailor the assessment to individual trait levels using computerized adaptive testing (CAT) [10]. In an IRT-based CAT, the successive items are chosen based on given answers to previous items. Because of this tailored administration of items, individuals only respond to a minimal number of relevant items.

To develop the PROMIS Pain Interference item bank, items from existing PROMs were collected, combined and revised and new items were developed to ensure the full range of the construct was covered [11]. PROMIS item banks and CATs have been shown to have strong content validity, good responsiveness and other desirable psychometric properties, and have the potential to be implemented worldwide [12–15]. Furthermore, PROMIS scores are easier to interpret than traditional Patient-Reported Outcome Measures (PROMs) scores, because the PROMIS scores are expressed on a standardized *T*-score metric.

The Dutch-Flemish PROMIS Group translated 17 adult PROMIS item banks and 9 paediatric PROMIS item banks into Dutch-Flemish (to accommodate the Dutch-speaking part of Belgium in addition to those in The Netherlands), including the PROMIS Pain Interference item bank. Details of this work have been published [16,17].

The first aim of the current study was to calibrate the Dutch-Flemish PROMIS Pain Interference item bank based on responses to items by Dutch patients with chronic pain. The second aim was to evaluate the cross-cultural validity between scores on the Dutch-Flemish and the United States (US) PROMIS Pain Interference item bank. The third study aim was to evaluate the reliability and construct validity of the Dutch-Flemish PROMIS Pain Interference item bank scores.

## Methods

The study was approved by the local institutional review board (Medical Ethical Committee Slotervaart hospital and Reade). To be eligible, patients had to provide written informed consent.

### Study participants

For this study, 2808 patients from the Amsterdam Pain (AMS-PAIN) cohort were invited to participate. The AMS-PAIN cohort consists of chronic pain patients who have been registered since September 2010 in Reade; an outpatient secondary care center for rheumatology and rehabilitation in the Netherlands. To be eligible, patients had to have at least one chronic pain condition for at least three months prior to participating in the study and had to be 21 years or older.

For evaluating the cross-cultural validity (or measurement equivalence) of the Dutch-Flemish versus the US PROMIS Pain Interference item bank, data from the US PROMIS Pain Interference American Chronic Pain Association (ACPA) sample was used. The ACPA sample consists of 967 patients with chronic pain who completed the PROMIS Pain Interference item bank [5]. All ACPA chronic pain patients met study eligibility criteria of being 21 years or older and having at least one chronic pain condition for at least three months prior to participating in the US PROMIS Wave 1 study [5].

### Procedures

Patients from the AMS-PAIN cohort were invited by e-mail or letter, to fill in a web-based (digital) or paper-and-pencil (paper) questionnaire that included, among other measures, the full Dutch-Flemish PROMIS Pain Interference item bank. For the digital questionnaire, patients received personal login codes. Patients who were unable to complete the digital questionnaire were asked to complete the paper version.

### Measures

The questionnaire included the full Dutch-Flemish PROMIS Pain Interference item bank. The translation of the US PROMIS Pain Interference item bank into Dutch-Flemish was performed by Functional Assessment of Chronic Illness Therapy multilingual translation (FACITtrans) using standardized methodology and approved by the PROMIS Statistical Center [16,18]. This translation included multiple forward and back translations, independent reviews and pilot testing with cognitive debriefing among 70 Dutch and Flemish adults [16]. The Dutch-Flemish PROMIS Pain Interference item bank contains 40 items covering a wide range of pain interferences content [5]. The time frame is the past 7 days. There are three different 5-point likert response scales: 1) not at all/a little bit/somewhat/quite a bit/very much; 2) never/rarely/sometimes/often/always; 3) never/once a week or less/once every few days/once a day/every few hours [5]. Demographic information also was collected (i.e. age, gender, country of birth, educational level).

In addition, the questionnaire contained five legacy instruments including the pain intensity item (Global07) from the Dutch-Flemish PROMIS Global Health item bank (an 11-point numeric rating scale (NRS) with 0 = ‘no pain’ and 10 = ‘worst pain imaginable’) [19]. Four reliable and valid condition-specific instruments were also included. The “Neck Disability Index” (NDI) consists of 10 items measuring self-reported pain intensity and the influence of neck pain on daily activities, with a total score ranging from 0 to 50 [20,21]. Evidence has accumulated for the reliability and validity of the NDI within Dutch patients with chronic neck pain [21–24]. The “Disabilities of the Arm, Shoulder and Hand” (DASH) questionnaire was used for patients with chronic shoulder pain. The DASH consists of 30 items measuring disabilities of the upper extremities, with a total score ranging from 0 to 100 [25,26]. DASH scores have demonstrated good reliability and validity in Dutch patients with a variety of disorders of the upper limb [25,27–29]. The “Roland Morris Disability Questionnaire” (RMDQ) consists of 24 items measuring disabilities as a result of chronic back pain, with a total score ranging from 0 to 24 [30,31]. RMDQ scores have demonstrated good reliability and validity within Dutch patients with chronic low back pain [30,32–34]. The fourth condition-specific legacy instrument was the “Fibromyalgia Impact Questionnaire” (FIQ), used for patients with fibromyalgia. The FIQ consists of 20 items measuring physical disabilities as a result of fibromyalgia, with a total score ranging from 0 to 100 [35,36]. FIQ scores have demonstrated moderate to good reliability and validity among Dutch patients with fibromyalgia [36,37]. For each legacy instrument higher scores indicate more intensity, disability or impact.

### Statistical analysis

#### Calibration of the Dutch-Flemish PROMIS Pain Interference item bank

The psychometric analyses were conducted using the PROMIS analysis plan [10]. Similar analyses were done as for the calibration of the Dutch-Flemish PROMIS Pain Behavior item bank [38]. To evaluate the measurement properties of the pain interference items, IRT based analyses were used. IRT models estimate the relationship between an item response category and the level of the measured construct, in this study the level of pain interference. Before calibrating the item parameters of the Dutch-Flemish PROMIS Pain Interference item bank, the three IRT assumptions, unidimensionality, local independence and monotonicity, were evaluated [10].

Unidimensionality was examined using Confirmatory Factor Analyses (CFA) in which all items were hypothesized to load on a single factor. The analysis was performed using the R-package (version 3.0.1) Lavaan (version 0.5–16), and model fit was evaluated based on the Comparative Fit Index (CFI), Tucker Lewis Index (TLI) and Root Means Square Error of Approximation (RMSEA) [39]. We used recommended criteria for unidimensionality (CFI >0.95, TLI >0.95, and RMSEA <0.06) [10]. Furthermore, unidimensionality was considered sufficient when the first factor accounts for at least 20% of the variability and when the ratio of the variance explained by the first to the second factor was greater than 4 [10,40]. This was examined with exploratory factor analysis (EFA).

Another IRT assumption is local independence, which means that after controlling for the dominant factor, there should be no significant covariance among item responses. Local dependency was evaluated by examining the residual correlation matrix resulting from the single factor CFA. Residual correlations greater than 0.2 were considered indicators of possible locally dependence [10]. In addition, local independence was studied using Yen’s Q3 statistic [41]. This statistic calculates the residual item scores under the graded response model (GRM) and correlates these among items. Cohen's rules of thumb were used for correlation effect sizes [42]. In this, Q3 values between 0.24 and 0.36 are moderate deviations, and values of 0.37 and greater represent large deviations. The impact of local dependency on IRT parameter estimates was evaluated, by removing the locally dependent items one by one and examining changes in the IRT parameters of the remaining items [10].

A third IRT assumption is monotonicity, in which the probability of endorsing a higher item response category should increase (or at least not decrease) with increasing levels of the underlying construct. Monotonicity of the Dutch-Flemish PROMIS Pain Interference items was evaluated by fitting a non-parametric IRT model, using Mokken scaling in the R-package Mokken [43,44]. This model yields nonparametric IRT response curve estimates, shows the probabilities of endorsing response categories and can be visually inspected to evaluate monotonicity.

After evaluation of the IRT assumptions, a GRM was fit to the item response data using the R-package Ltm [45,46]. The GRM models two item parameters, the item thresholds and the item slope [10]. Item threshold parameters indicate item difficulty, locate items along the measured trait, and show the coverage across the pain interference continuum. The item slope parameter represents the discriminative ability of the items, with higher slope values indicating better ability to discriminate between adjoining values on the construct.

To assess the fit of the GRM and the degree in which possible misfit affects the IRT model, S-X^2^ statistic was used [47]. This statistic compares the observed and expected response frequencies under the estimated IRT model, and quantifies the differences between the observed and expected response frequencies. Items with a S-X^2^ p-value of less than 0.001, were considered to have poor fit [10,47].

#### Differential item functioning within the Dutch AMS-PAIN sample

Differential item functioning (DIF) analyses are used to examine if people from different groups (e.g. age or gender) with the same level of trait (in this study the same level of pain interference) have different probabilities of giving a certain response to an item [10,48,49]. There are two kinds of DIF: uniform and non-uniform [10,48,49]. Uniform DIF exists when the DIF is consistent, with the same magnitude of DIF across the entire range of the trait. Non-uniform DIF exists when the magnitude or direction of DIF differs across the trait. DIF was evaluated with use of the R package Lordif (version 0.2–2) using ordinal logistic regression models with a McFadden’s pseudo R^2^ change of 2% as critical value [10,50,51]. In this portion of the study, we used this method to evaluate DIF *within* the Dutch AMS-PAIN sample based on age (Median split: under 50 years vs. 50 years and over), gender (male vs. female), and administration mode (digital vs. paper).

#### Reliability

Reliability within IRT is conceptualized as “information”, in which the fact that measurement precision can differ across levels of the measured trait (*θ* = Theta) is taken into account. The relationship between information and standard error (SE) is defined by the formula: $SE(\theta )=\frac{1}{\sqrt{\text{I}(\theta )}}$, where SE is the standard error of estimated *θ*, I is information, and *θ* is the estimated trait level (ranging from no or mild pain interference to high levels of pain interference) [5,11]. The formula indicates that increased scale information is related to smaller SE’s and, therefore, greater measurement precision. Using the calculated SEs, plots were overlaid showing SE (as an indicator of reliability) across the score range of the 4-item short form (v1.0.4a), the 8-item short form (v1.0.8a), the 8-item simulated CAT (always 8 items, no other stopping rules), and the total item bank. The 8-item simulated CAT was conducted with use of the R-package catR (version 3.4) [52]. The IRT theta scores of the Dutch AMS-PAIN sample were transformed into T-scores anchored on the US item parameters cue sheet of the US PROMIS Pain Interference item bank [5]. In which T-score 50 represents the average score of the general US population, with a standard deviation of 10. For the total item bank Cronbach’s alpha was calculated.

#### Cross-cultural validity

Differences in descriptive characteristics between the Dutch AMS-PAIN patients and the US ACPA patients were evaluated with use of independent samples t-tests and Chi square-tests, for continuous and categorical variables respectively.

For the evaluation of cross-cultural validity of the Dutch-Flemish PROMIS Pain Interference item bank versus the US PROMIS Pain Interference item bank, DIF for language (Dutch vs. English) was analysed, with use of the R package Lordif (version 0.2–2), using ordinal logistic regression models with a McFadden’s pseudo R^2^ change of 2% as critical value [10,50,51]. When items were flagged as potential DIF items, the wABC effect size index was computed [53]. Furthermore, the impact of DIF was examined by plotting item characteristic curves (ICC) (not shown) and test characteristic curves (TCC). The TCC plots showed the scores for all 40 Pain Interference items (ignoring DIF), and the scores for only the items having DIF [51].

#### Construct validity

Construct validity of the Dutch-Flemish PROMIS Pain Interference item bank was evaluated by correlating the T-scores of the Dutch-Flemish PROMIS Pain Interference item bank to the (total) scores on the legacy instruments (Dutch-Flemish PROMIS Global Health pain intensity item score, NDI, DASH, RMDQ, and FIQ). Construct validity was evaluated using Pearson correlations. We hypothesized that the Dutch-Flemish PROMIS Pain Interference item bank scores would have high correlations (*r* >0.50) with all the legacy instruments.

## Results

### Participants

Of the 2808 invited patients of the Dutch AMS-PAIN cohort, 1140 responded to the questionnaire (response rate 40.6%). No differences were found between responders and non-responders on age, gender, country of birth, or education level. Among the 1140 respondents, 29 patients were excluded because they did not give informed consent and 26 patients responded to none of the items of the Dutch-Flemish PROMIS Pain Interference item bank, leaving N = 1085 patients. Because the GRM analyses can accommodate incomplete data, all 1085 were used for the IRT calibration. All other analyses were based on responses of the 973 patients with complete data.

The demographic characteristics of the Dutch AMS-PAIN sample and the US ACPA chronic pain sample are summarized in Table 1. Of the AMS-PAIN patients, 78% (n = 846) were female and the average age (SD) was 49 years (13) with a range from 21 to 85. Fifty-seven percent (n = 621) of these were born in the Netherlands, and 82% had at least a high school degree. Of the AMS-PAIN patients, 83% (n = 891) indicated that the duration of their pain was more than 2 years, and the average pain intensity on a NRS (SD) was 6.6 (2). Patients reported having chronic low back pain (71%), chronic neck or shoulder pain (70%), fibromyalgia (35%), chronic widespread pain (47%), migraine or other chronic headache (35%), and osteoarthritis (35%). Twelve percent of the chronic pain patients reported having rheumatoid arthritis and 2% reported cancer. No differences were found in age, gender or pain intensity between the Dutch AMS-PAIN sample and the US ACPA sample. However, there were some differences in educational levels, pain duration and type of chronic pain condition. Slightly more Dutch AMS-PAIN patients reported pain duration of 1–2 years. The US ACPA sample was more educated with 97% reporting high school education or more, while in the Dutch AMS-PAIN sample 82% reported high school education level or more.

**Table 1:** Demographic characteristics of the Dutch AMS-PAIN sample (n = 1085) and the US ACPA sample (n = 967).

	Dutch chronic pain sample	US chronic pain sample
**Age**		
Mean (SD)	49 (13)	48 (11)
Range	21–85	21–86
**Gender** n (%)		
Male	239 (22)	182 (19)
Female	846 (78)	780 (81)
**Country of birth** n (%)		
Netherlands	621 (57)	-
Other	464 (43)	-
**Social status** * n (%)		
Single	373 (34)	-
Married or living together	578 (53)	-
Living apart together	57 (5)	-
Living with parents	24 (2)	-
Other	61 (6)	-
**Educational level** n (%)		
Less than High School degree	188 (18)	23 (3) ^^
High School degree	147 (15)	157 (16)
Some college	410 (41)	452 (47)
College degree	46 (5)	214 (22) ^^
Advanced degree	218 (21)	114 (12) ^^
**Employment status** * n (%)		
Full-time	173 (16)	-
Part-time	271 (25)	-
Student	42 (4)	-
Unpaid, volunteer, household	164 (15)	-
Retired	88 (8)	-
Unemployed	194 (18)	-
Other	219 (20)	-
**Social benefits** * n (%)		
Sick listed	235 (22)	-
Disability benefit	244 (19)	-
Unemployment benefit	90 (8)	-
Other	141 (13)	-
No social benefit	381 (35)	-
**Duration of pain** n (%)		
3–6 months	14 (1)	15 (2)
6–12 months	32 (3)	38 (4)
1–2 years	138 (13)	65 (7) ^^
2–5 years	316 (29)	234 (25)
>5 years	575 (54)	577 (62)
**Type of chronic pain condition** * n (%)		
Migraine and/or other ‘daily’ headache	381 (35)	209 (22)^^
Rheumatoid arthritis	135 (12)	59 (6)^^
Osteoarthritis	380 (35)	195 (20)^^
Pain related to cancer	19 (2)	8 (0.8)^
Lower back pain	775 (71)	533 (55)^^
Neck or shoulder pain	760 (70)	447 (46)^^
Fibromyalgia	378 (35)	338 (35)
Chronic widespread pain	508 (47)	-
Other neuropathic pain (nerve damage)	222 (21)	370 (38)^^
Other	491 (45)	298 (31)^^
No chronic pain condition	6 (0.6)	1 (0.1)^^
**T-score**		
Mean (SD)	64.1 (6.8)	68.6 (4.9)
Range	40.1–84.0	53.0–90.0^^
**Legacy instruments** mean (SD)		
PROMIS Global Health Pain intensity (n = 1033)	6.6 (2)	6.6 (2)
NDI (n = 399)	25 (9)	-
DASH (n = 390)	46 (20)	-
RMDQ (n = 648)	13 (6)	-
FIQ (n = 295)	60 (18)	-

PROMIS Global Health Pain Intensity (0–10); NDI = Neck Disability Index (0–50); DASH = Disabilities of the Arm, Shoulder and Hand (0–100); RMDQ = Roland Morris Disability Questionnaire (0–24); FIQ = Fibromyalgia Impact Questionnaire (0–100). Higher scores indicate more intensity, disability or impact

* multiple answers were allowed

^ p<0.05

^^p<0.001.

### Calibration of the Dutch-Flemish PROMIS Pain Interference item bank

The CFA results indicated good fit to a unidimensional model. The CFI was 0.986 and the TLI was 0.986, which are above the criterion of >0.95 [10]. However, the RMSEA was 0.159, which is somewhat larger than the criterion of <0.06. For the 8-item short form (v1.0.8a), the CFI was 0.996, the TLI 0.995 and the RMSEA 0.161. The first factor in EFA accounted for 66% of the variance, and the second factor accounted for 5% of the variance; hence the ratio of the variance explained by the first to the second factor is 13, which favourably exceeds the published criterion of 4 [10]. Based on these results, it was concluded that the Dutch-Flemish PROMIS Pain Interference items share a single common factor, and are sufficiently unidimensional for modelling using the GRM.

Examining the residual correlation matrix showed a small number of local dependent item pairs. Twenty-five out of the 780 items pairs (3.2%) had residual correlations greater than 0.2. Yen’s Q3 statistic values of 49 of the 780 item pairs (6.3%) indicated at least a moderate deviation of model fit. The item pairs with the greatest dependency were PAININ42 *(“How often did pain prevent you from standing for more than one hour*?*”)*–PAININ47 *(“How often did pain prevent you from standing for more than 30 minutes*?*”)* with residual correlation of 0.77, and PAININ50 *(“How often did pain prevent you from sitting for more than 30 minutes*?*”)*–PAININ55 *(“How often did pain prevent you from sitting for more than one hour*?*”)* with residual correlation of 0.70. These items were removed one by one and then the impact on item parameters of the remaining items was evaluated. After removal of item 42 or 47, the mean difference in item thresholds of the remaining items was 0.004, the mean difference in the slope parameters was 0.01, and the residual correlation of the next highest item pair was 0.254. The mean difference in item thresholds after removal of item 50 or 55 was 0.15, the mean difference in slope parameters was 0.12, and the residual correlation of the next highest item pair was 0.265. These results suggest minimal impact of local dependence.

The Mokken scalability coefficient of the full pain interference item bank was 0.63, suggesting strong scalability according to published criteria [43,44,54]. All of the items had a scalability coefficient that was higher than the lower bound of 0.30. Based on these results, it was concluded that the Dutch-Flemish PROMIS Pain Interference items met the assumption of monotonicity.

Table 2 summarizes the IRT item parameters of the Dutch-Flemish PROMIS Pain Interference items. The item threshold parameters ranged from -3.04 to 3.44. The item slope parameters ranged from 0.95 to 3.02. The item with lowest discrimination parameter was PAININ54 *(“How often did pain keep you from getting into a standing position*?*”)*, and the item with the highest discrimination parameter was PAININ31 *(“How much did pain interfere with your ability to participate in social activities*?*”)*.

**Table 2:** IRT item characteristics for the Dutch-Flemish PROMIS Pain Interference item bank.

			Category threshold					Item fit statistics	
Item code	Item	Slope a	B1	B2	B3	B4	Mokken’s H	S-X^2^	Prob X^2^
PAININ1	How difficult was it for you to take in new information because of pain?	1.69	-1.63	-0.47	0.61	1.91	0.62	40.00	0.470
PAININ3	How much did pain interfere with your enjoyment of life? ◊	2.35	-2.11	-0.89	0.07	1.42	0.67	41.55	0.403
PAININ5	How much did pain interfere with your ability to participate in leisure activities?	2.55	-2.25	-1.11	-0.17	1.12	0.69	56.74	0.042
PAININ6	How much did pain interfere with your close personal relationships?	2.43	-1.39	-0.45	0.47	1.80	0.67	39.66	0.486
PAININ8	How much did pain interfere with your ability to concentrate?	1.67	-2.06	-0.89	0.22	1.67	0.61	25.67	0.962
PAININ9	How much did pain interfere with your day to day activities? * ◊	2.01	-2.94	-1.45	-0.27	1.45	0.66	29.87	0.879
PAININ10	How much did pain interfere with your enjoyment of recreational activities?	2.63	-2.15	-0.96	-0.13	1.21	0.69	34.88	0.670
PAININ11	How often did you feel emotionally tense because of your pain?	1.91	-2.07	-0.99	0.06	1.35	0.65	38.22	0.550
PAININ12	How much did pain interfere with the things you usually do for fun? ◊	2.56	-2.24	-1.00	-0.12	1.25	0.69	39.27	0.503
PAININ13	How much did pain interfere with your family life? ◊	2.22	-1.68	-0.72	0.26	1.71	0.66	75.62	0.0006#
PAININ14	How much did pain interfere with doing your tasks away from home (e.g., getting groceries, running errands)?	2.21	-1.65	-0.70	0.13	1.40	0.67	58.31	0.031
PAININ16	How often did pain make you feel depressed?	1.55	-1.61	-0.62	0.78	2.54	0.60	35.61	0.668
PAININ17	How much did pain interfere with your relationships with other people?	2.39	-1.54	-0.50	0.46	1.84	0.67	38.69	0.529
PAININ18	How much did pain interfere with your ability to work (include work at home)?	2.29	-2.41	-1.30	-0.50	0.78	0.68	50.47	0.124
PAININ19	How much did pain make it difficult to fall asleep?	1.40	-2.00	-0.91	0.10	1.27	0.58	45.68	0.248
PAININ20	How much did pain feel like a burden to you?	2.23	-3.04	-1.57	-0.56	0.83	0.68	31.83	0.818
PAININ22	How much did pain interfere with work around the home? * ◊	2.21	-2.57	-1.43	-0.48	1.04	0.67	30.76	0.853
PAININ24	How often was pain distressing to you? ^	1.48	-2.11	-0.99	0.46	2.31	0.59	49.22	0.150
PAININ26	How often did pain keep you from socializing with others?	2.25	-2.00	-1.10	0.17	2.10	0.68	46.81	0.213
PAININ29	How often was your pain so severe you could think of nothing else?	1.83	-1.66	-0.57	0.73	2.55	0.64	29.25	0.895
PAININ31	How much did pain interfere with your ability to participate in social activities? * ◊	3.02	-1.92	-0.83	0.01	1.41	0.70	38.61	0.533
PAININ32	How often did pain make you feel discouraged? ^	1.77	-2.05	-1.04	0.38	2.37	0.63	53.84	0.071
PAININ34	How much did pain interfere with your household chores? * ◊	2.13	-2.63	-1.44	-0.46	1.06	0.67	24.70	0.973
PAININ35	How much did pain interfere with your ability to make trips from home that kept you gone for more than 2 hours?	2.47	-1.37	-0.65	0.12	1.23	0.68	59.18	0.026
PAININ36	How much did pain interfere with your enjoyment of social activities? ◊	2.66	-1.92	-0.90	-0.08	1.26	0.69	35.55	0.671
PAININ37	How often did pain make you feel anxious?	1.37	-1.75	-0.66	0.84	2.58	0.56	47.58	0.191
PAININ38	How often did you avoid social activities because it might make you hurt more?	1.92	-1.67	-0.83	0.26	1.95	0.64	49.50	0.144
PAININ40	How often did pain prevent you from walking more than 1 mile?	1.28	-2.12	-1.23	-0.24	1.03	0.54	58.90	0.027
PAININ42	How often did pain prevent you from standing for more than one hour?	1.20	-2.63	-1.77	-0.82	0.63	0.54	73.47	0.001#
PAININ46	How often did pain make it difficult for you to plan social activities?	2.31	-1.93	-1.04	0.04	1.58	0.69	38.31	0.546
PAININ47	How often did pain prevent you from standing for more than 30 minutes?	1.21	-2.26	-1.39	-0.17	1.20	0.54	63.38	0.011
PAININ48	How much did pain interfere with your ability to do household chores?	2.20	-2.38	-1.19	-0.33	1.09	0.68	30.49	0.861
PAININ49	How much did pain interfere with your ability to remember things?	1.48	-1.51	-0.31	0.71	2.05	0.59	38.31	0.547
PAININ50	How often did pain prevent you from sitting for more than 30 minutes?	1.42	-1.53	-0.50	0.62	2.00	0.58	44.04	0.304
PAININ51	How often did pain prevent you from sitting for more than 10 minutes?	1.28	-0.73	0.44	1.82	3.44	0.56	34.79	0.703
PAININ52	How often was it hard to plan social activities because you didn't know if you would be in pain?	1.82	-1.50	-0.70	0.24	1.73	0.64	30.34	0.865
PAININ53	How often did pain restrict your social life to your home?	2.17	-1.61	-0.79	0.26	1.93	0.68	36.70	0.620
PAININ54	How often did pain keep you from getting into a standing position?	0.95	-0.86	-0.01	0.91	1.76	0.48	50.43	0.125
PAININ55	How often did pain prevent you from sitting for more than one hour?	1.32	-1.65	-0.77	0.21	1.77	0.56	45.82	0.243
PAININ56	How irritable did you feel because of pain?	1.67	-2.29	-0.72	0.41	1.78	0.61	40.50	0.448

^ uniform DIF due to language (Dutch versus English). Activity is relatively faster endorsed in US chronic pain patients.

* item included in the 4-item short form (V1.0.4a).

◊ item included in the 8-item short form (V1.0.8a).

# p≤0.001.

The probability values for the S-X^2^ statistics ranged from 0.0006 to 0.9725. Based on the S-X^2^ p-value of less than 0.001, only 2 out of 40 items (PAININ13 and PAININ42) were found to misfit the GRM.

### Differential item functioning by language

None of the Dutch-Flemish PROMIS Pain Interference items were flagged for DIF for age, gender, or administration mode.

### Reliability

**Fig 1:**
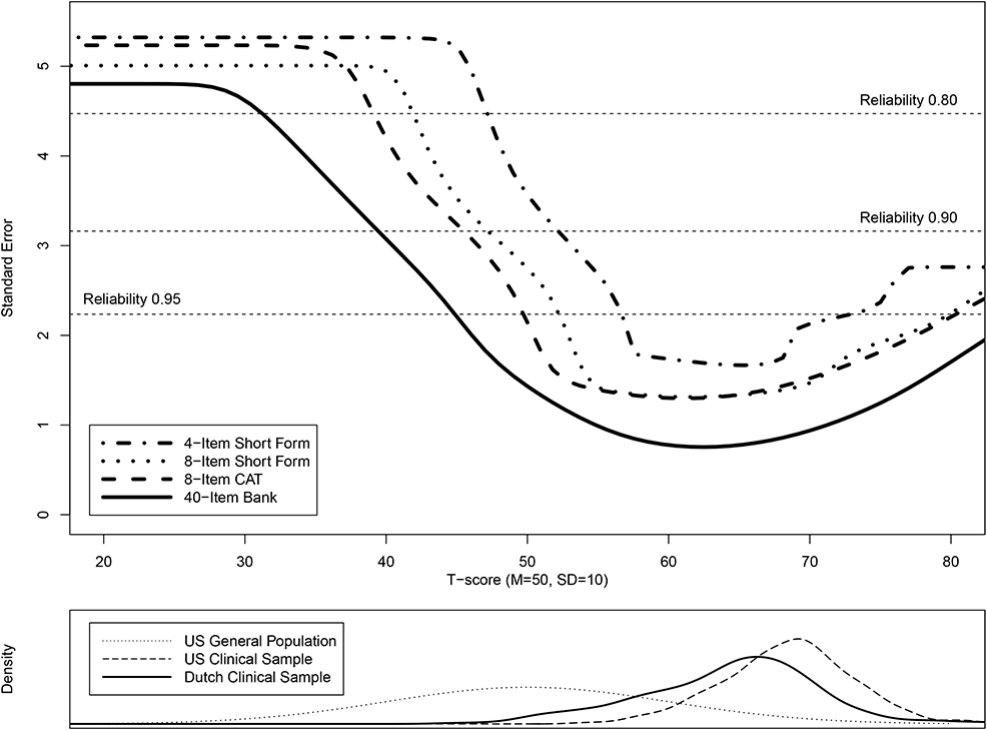
Standard errors across the range of the Dutch-Flemish PROMIS Pain Interference T-scores. Upper plot shows the standard errors of the Dutch-Flemish PROMIS Pain Interference 4-item short form (v1.0.4a), the 8-item short form (v1.0.8a), the 8-item simulated CAT, and the total item bank. Lower plot shows the distribution of the Dutch AMS-PAIN (Dutch clinical) sample, the US ACPA (US clinical) sample and the US Wave1 sample (US general population) along the T-score scale.

As shown in Table 1, the mean T-score for the overall Dutch-Flemish PROMIS Pain Interference AMS-PAIN sample was 64.1 (SD = 6.8), with a range from 40.1 to 84.0. The corresponding means for the US ACPA (clinical) sample and general US (community) sample were 68.6 (SD = 4.9) and 50 (SD = 10). Fig 1 shows the distributions of the T-score from the three samples. Fig 1 also includes plots of standard errors across the range of the Dutch-Flemish PROMIS Pain Interference T-scores, for the 4-item short form (v1.0.4a), the 8-item short form (v1.0.8a), the 8-item simulated CAT, and the total item bank. Between a T-score of 40 and 82 the reliability of the total item bank is greater than 0.90. Between a T-score of 44 and 82, where 96% of the Dutch AMS-PAIN sample is located, the reliability is even higher (> 0.95). The 8-item CAT and 8-item short form show similar results, in which the reliability was 0.90 or greater across the T-score range of 45 to 82. The plot also indicates that the 8-item short form performs slightly better than the 8-item CAT at very low levels of pain interference (T-score <37). The Cronbach’s alpha estimate for the total item bank was 0.98. These results indicate good internal consistency of the Dutch-Flemish PROMIS Pain Interference item bank.

### Cross-cultural validity

The analysis of DIF for language, flagged 2 items with some level of uniform DIF (see Table 2): PAININ24 (R^2^ = 0.052, wABC = 0.597) and PAIN32 (R^2^ = 0.025, wABC = 0.423). For both items (PAININ24: *“How often was pain distressing to you*?*”* and PAININ32: *“How often did pain make you feel discouraged*?*“*) the Dutch patients were more likely to endorse lower response categories compared to the US patients who were at the same level of the trait.

**Fig 2:**
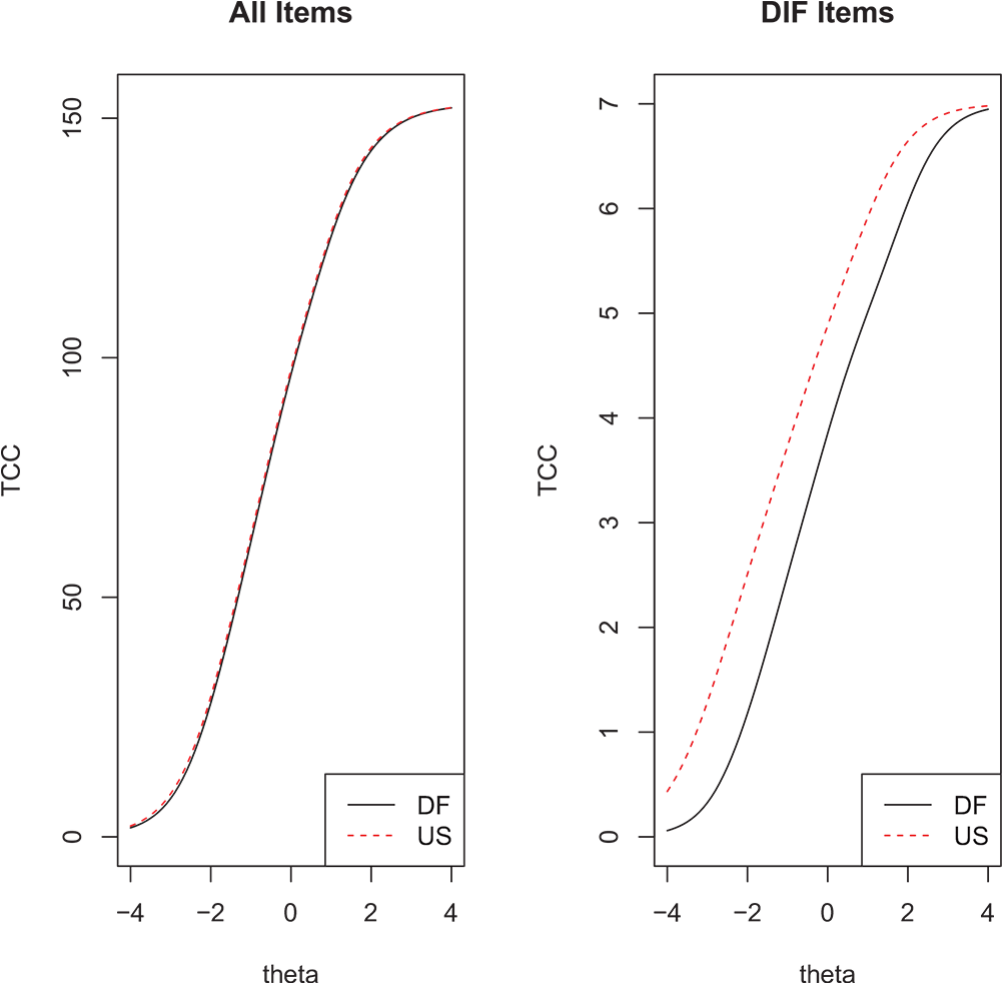
The overall impact of DIF for language on the test characteristic curves (TCC). The TCC shows the relation between the total item scores (y-axis) and theta (x-axis). Left graph shows the TCC for all 40 Dutch-Flemish (DF) and United States (US) PROMIS Pain Interference items (ignoring DIF); the right graph shows the TCC for just the 2 items having DIF.

The overall impact of DIF for language on the TCC is shown in Fig 2. The left graph shows the TCC for all 40 Pain Interference items (ignoring DIF), and the right graph shows the TCC for just the 2 items having DIF. These curves show that the Pain Interference total score is only slightly lower for Dutch patients than for US patients, indicating minimal impact of DIF by language. In fact, as the right hand figure shows that accounting for DIF in the two flagged items would change the score on the full bank by less than a half point.

### Construct validity

Pearson correlation coefficients indicate the relations between T-scores of the Dutch-Flemish PROMIS Pain Interference item bank and those of the legacy instruments. As expected, the Dutch-Flemish PROMIS Pain Interference item bank correlated highly (*r* > 0.50) with all legacy instruments (PROMIS Global Health Pain intensity, 0.75; NDI, 0.74; DASH, 0.71; RMDQ, 0.62; and, FIQ, 0.75).

## Discussion

The aim of current study was to calibrate the Dutch-Flemish PROMIS Pain Interference item bank in Dutch patients with chronic pain and to evaluate cross-cultural validity of the Dutch-Flemish compared to the US PROMIS Pain Interference item bank. The reliability and construct validity of the Dutch-Flemish PROMIS Pain Interference item bank were evaluated. The results supported the unidimensionality, model fit, and breadth of coverage of the Dutch-Flemish pain interference bank. Furthermore, the analyses showed no evidence for DIF due to age, gender, or administration mode; and, scores exhibited good cross-cultural validity, reliability, and construct validity. This study is the first calibration study of the Dutch-Flemish PROMIS Pain Interference item bank.

The Dutch-Flemish PROMIS Group aims to improve the measurement of patient reported outcomes in the Netherlands and Flanders (the Dutch-speaking part of Belgium) by providing and supporting the implementation of IRT-based, efficient, highly reliable and valid PROMIS item banks and CATs [16]. PROMIS item banks and CATs have better content validity compared to traditional PROMs [13]. PROMIS item banks are based on a well-developed conceptual model with clearly defined unidimensional constructs and have been developed using extensive qualitative research with patients [15]. PROMIS item banks show good measurement properties; they have small measurement errors and show better responsiveness compared to more traditional PROMs [12,14,15]. This makes the use of PROMIS item banks in daily clinical practise more suitable than traditional PROMs. Increased responsiveness results in reduction of sample sizes needed in clinical studies [12,14]. Through the use of IRT-based methods, PROMIS item bank and CAT scores approximate an interval scale instead of an ordinal scale, and therefore are easier to interpret than scores of more traditional PROMs [55,56]. The PROMIS scores are expressed on a common standardized T-score metric, and because they are calibrated using an IRT model, the T-scores can be estimated even if people do not respond to the same items, for instance when using CAT. The use of CAT has great advantages compared to more traditional paper questionnaires; CATs are tailored to the patients’ ability and therefore more efficient and precise [55,56].

The analyses of the IRT assumptions show that the required assumptions of unidimensionality and monotonicity are met, but there is some local dependence. The CFA results, CFI as well the TLI supported unidimensionality. The RMSEA was beyond the criterion of <0.06, but RMSEA values tend to be elevated when the number of items is large [57]. Furthermore, Miles and Shevlin (2007) indicate that the RMSEA alone is not meaningful; you have to consider the fit indices (CFI, TLI and RMSEA) as a whole, together with the sample size and the reliability of the measurement to determine the model fit [58]. Therefore, given the high CFI and TLI, the large sample size and high reliability of the item bank, the high RMSEA in the current study is of little concern. The results of local dependence suggest that a certain amount of local dependence is present. This could possibly influence the T-scores computed with a CAT. However, these local dependence results are only based on the analyses of the Dutch AMS-PAIN sample, and before we make decisions on removing items from the Dutch-Flemish PROMIS Pain Interference item bank, we need to reproduce the analyses in a Dutch general population sample. Therefore, it was decided not to remove items from the item bank at this moment. Until we reproduce the analyses in a Dutch general population sample, we can prevent that items that show local dependence are both being administered in a CAT. The calibration analyses of the Dutch-Flemish PROMIS Pain Interference items, show that the range of the item threshold parameters indicates good coverage across the range of the pain Interference construct. Furthermore the item threshold parameters show which items are most useful for measuring different levels of Pain Interference, which is required for the selection process of items in a CAT.

No items were flagged for DIF with respect to gender, age and administration mode. Therefore, the Dutch-Flemish PROMIS Pain Interference items and scores can be used across patients that differ in gender and age, and differ in the way of completing the item bank (digital or paper).

Although the response rate in this study was only 40.6%, the large sample size of 1085 patients is reassuring. When comparing the Dutch AMS-PAIN sample with the US ACPA sample, no differences were found in age, gender and pain intensity. However, the differences in educational level are noteworthy, where the US ACPA patients were more educated than the Dutch AMS-PAIN patients (97% vs 82% reporting high school education or more).

The evaluation of cross-cultural validity of the Dutch-Flemish PROMIS Pain Interference item bank versus the US PROMIS Pain Interference item bank identified evidence of DIF for language across 2 out of the 40 items. However, DIF had a minimal impact on the item scores. Therefore we conclude that the cross-cultural item differences were negligible and that all items can be retained in the item bank. For both items showing DIF there are some potential translational improvements. Therefore, we recommend testing new (possibly better) translations of these two items in a future data collection.

The plot of the standard errors across the range of the Dutch-Flemish PROMIS Pain Interference T-scores shows that the 8-item short form performs slightly better than the 8-item CAT at very low levels of pain interference (T-score <37). This could possibly be explained by the item selection procedure used in the simulated CAT [59]. For estimating person’s T-score, the CAT starts in the middle of the trait-range (T-score = 50) asking the item with the highest information [59]. Because of this item selection procedure, possibly an 8-item CAT is too short to provide an accurate estimate of low T-scores [59]. However, the impact of this will only be minimal because there are almost no respondents with such low levels (T-score<37) of pain interference. Furthermore, the difference will disappear when using the CAT stopping rule of SE<0.3 (more commonly used method) instead of using a fixed number of items. Another explanation could be that the short forms include items covering the whole construct, where a CAT doesn’t because the items chosen in a CAT depend on the persons’ level of the construct. In this study, most patients were located at the higher level of the pain interference construct, through which items at the lower end of the pain interference construct have a lower probability of being administered in the simulated CAT

This study supports the construct validity of the Dutch-Flemish PROMIS Pain Interference item bank, in which the correlations between the Dutch-Flemish PROMIS Pain Interference item bank and the legacy instruments were high, as expected.

The Dutch-Flemish PROMIS Pain Interference item bank is ready to be used as an item bank or short form. A 4-item PROMIS Pain Interference short form was developed within PROMIS (v1.0.4a), including items with the highest information value. Furthermore, a 6-item (v1.0.6b) and an 8-item (v1.08a) PROMIS Pain Interference short form were developed within PROMIS. When selecting Dutch-Flemish PROMIS Pain Interference items for short forms, it would be preferable to select items without DIF. Fortunately, the two items showing DIF for language are not included in the PROMIS Pain Interference short forms.

The Dutch-Flemish PROMIS Pain Interference item bank is now calibrated in Dutch persons with chronic pain and ready for use. For the time being, we recommend to use US PROMIS Pain Interference item parameters and the US T-score metric, with T = 50 as mean T-score of the general US population as reference-point and on which the Dutch chronic pain sample is anchored with a mean T-score of 64.1. We recommend future analyses on data collected with the Dutch-Flemish PROMIS Pain Interference item bank in the general Dutch and Flemish population, and in patient groups with other health problems resulting in (chronic) pain. After data collection in the general Dutch and Flemish population the item bank needs to be recalibrated, and then a Dutch-Flemish T-score metric can be developed with a T = 50 as mean T-score of the general Dutch-Flemish population as reference-point. Also, it should then be decided if Dutch-Flemish specific item parameters are needed or whether the US item parameters can also be used in Dutch-Flemish patients. Furthermore, for future research it would be interesting to study DIF for other factors than age, gender, administration mode and language (e.g. medical diagnosis). It also would be interesting to evaluate the impact of DIF on the Dutch-Flemish PROMIS Pain Interference scores obtained by CAT, by comparing a CAT applying the Dutch-Flemish item parameters with a CAT applying the original US item parameters. The impact of DIF may be greater when using CAT as compared to using the total item bank, because a CAT uses only a small item set [10]. Another important step for future research and also for implementing the Dutch-Flemish PROMIS Pain Interference item bank, short forms and CAT, is to further improve the interpretability of the PROMIS metric. For example, the bookmarking method methodology, adapted from educational testing, could be used to develop cut scores for clinically meaningful category intervals [60]. Other methods should be applied to identify PROMIS Pain Interference score differences that represent minimal important changes [60].

In conclusion, this item calibration study found good cross-cultural and construct validity of the Dutch-Flemish PROMIS Pain Interference item bank. The item bank has the potential to improve the measurement of pain interference. The Dutch-Flemish PROMIS Pain Interference item bank and short forms are now available for clinical application in Dutch speaking persons with chronic pain and a Dutch-Flemish PROMIS Pain Interference CAT can now be developed, for the time being using US PROMIS Pain Interference item parameters.
